# Generation of a p21 Reporter Mouse and Its Use to Identify and Eliminate p21^high^ Cells In Vivo

**DOI:** 10.3390/ijms24065565

**Published:** 2023-03-14

**Authors:** Zimei Yi, Le Ren, Yu Wei, Siyi Chen, Jiechen Zhao, Jiayu Zhu, Junhua Wu

**Affiliations:** Department of Prosthodontics, Stomatological Hospital and Dental School of Tongji University, Shanghai Engineering Research Center of Tooth Restoration and Regeneration, Shanghai 200072, China

**Keywords:** p21, transgenic mouse, cellular senescence, senescence-associated secretory phenotype (SASP)

## Abstract

P21 and p16 have been identified as inducers of senescence. Many transgenic mouse models have been developed to target cells expressing high levels of *p16^Ink4a^* (p16^high^) and investigate their potential contribution to tissue dysfunction in aging, obesity, and other pathological conditions. However, the specific roles of p21 in various senescence-driven processes remain unclear. To gain a deeper understanding of p21, we built a p21-3MR mouse model containing a p21 promoter-driven module that allowed us to target cells with high *p21^Chip^* expression (p21^high^). Using this transgenic mouse, we monitored, imaged, and eliminated p21^high^ cells in vivo. We also applied this system to chemically induced weakness and found that the clearance of p21^high^ cells improved doxorubicin (DOXO)-induced multi-organ toxicity in mice. By recognizing p21 transcriptional activation spatially and temporally, the p21-3MR mouse model can be a valuable and powerful tool for studying p21^high^ cells to further understand senescence biology.

## 1. Introduction

Cellular senescence is a state of proliferation stasis induced by mitosis and carcinogenesis that is characterized by the expression of cyclin-dependent kinase inhibitors (CDKIs) [1], such as p21 and p16 [2,3,4]. Senescent cells also produce a distinctive proinflammatory secretion known as the SASP, characterized by the release of various cytokines, chemokines, growth factors, and other inflammatory mediators [5]. Recent research has revealed that p16^high^ and p21^high^ cells represent distinct senescent cell populations, exhibiting heterogeneity in the expression of senescence markers across different tissues [6,7]. For example, islet tissue exhibits higher levels of p16 expression, whereas adipose tissue showed higher levels of p21 [8].

Previous studies have focused on p16-based mice models to examine the causal role of p16^high^ cells under aging conditions in vivo [9]. However, it is important to note that p16^high^ cells constitute only a small fraction of senescent cells in vivo, and a high level of p16 expression does not necessarily indicate a senescent cell, as not all senescent cells exhibit high levels of p16 expression [10]. More research has highlighted the importance of p21 in aging-related diseases [11,12,13]. Specifically targeting p21^high^ cells in the adipose tissue of diabetic mice can relieve insulin resistance in obese mice. Focusing on p21^high^ cells can inhibit radiation-induced osteoporosis, but not on p16^high^ cells [14]. P21^high^ cells contribute to injury responses in osteoarthritis through IL-17, whereas p16^high^ cells are not involved in the progression of osteoarthritis [15,16]. In light of these findings, it is evident that the p21 cell population plays a valuable and unique role, as described above. However, current p21-based mouse models are relatively homogenous and have limited utility. There is a need to create a multi-functional tool to investigate the role and underlying mechanisms of p21^high^ cells and senescence heterogeneity as well as to develop novel senolytic therapies [17].

Taking advantage of this, we created a new transgenic reporter mouse model, p21-3MR, that expressed a trimodal reporter protein (3MR) under the control of the endogenous p21 promoter within the *cyclin-dependent kinase inhibitor 1A*(*Cdkn1a*) gene locus to interrogate endogenous p21 promoter activity. DOXO, an anthracycline-based chemotherapeutic agent, can induce high p21 expression and promote the accumulation of cells with elevated p21 levels [18,19]. Then p21^high^ cells can be selectively killed by Ganciclovir (GCV) [20]. GCV has high affinity for HSV-TK and works by converting it into a toxic DNA strand terminator that causes genomic and mitochondrial DNA breaks. In our experiments, the 3MR containing HSV-TK was placed after the p21 promoter, so that only cells with high p21 promoter activity could bind to GCV and thus cause cell death. A combination of DOXO and GCV can serve as a means of verifying whether the p21 model can be successfully modeled [21].

DOXO is also used as one of the most common chemotherapeutic agents for the treatment of solid tumors, leukemias, and lymphomas [22,23]. However, DOXO exhibits deteriorating effects on healthy tissues. An analysis of the transcriptome indicated that cardiotoxicity induced by acute DOXO treatment was associated with dysregulation of the expression of genes associated with the p53-p21 pathway [24]. Although models of DOXO-induced cellular senescence have been established in vitro, the association between DOXO-mediated organ toxicity and cellular senescence, particularly p21, has not been precisely described to date. Our study shows that DOXO causes widespread structural degeneration of tissue, as well as inflammatory infiltration throughout the body. Interestingly, these effects appear to correlate with the expression of p21, as administration of GCV drugs to remove p21 cells improved tissue morphology in some tissues.

Based on our findings, we suggest that the p21-3MR mouse model may prove to be a valuable tool in the study of p21^high^ cells and could also open up exciting possibilities for studying senescent cell heterogeneity.

## 2. Results

### 2.1. Generation of the p21-3MR Mouse Model

**Figure 1:**
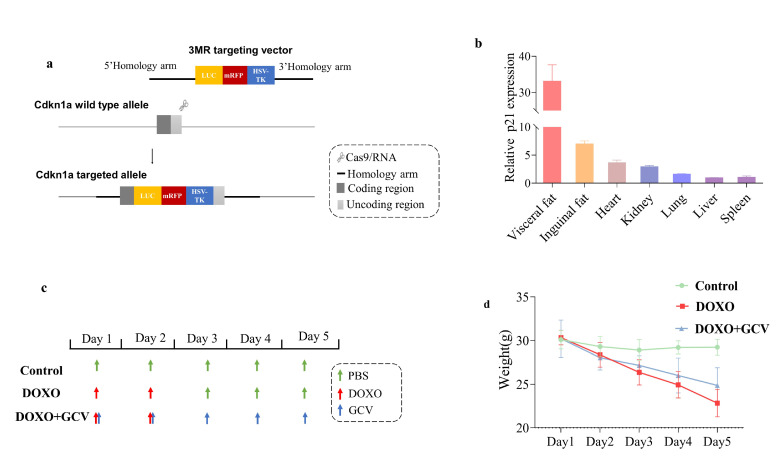
Generation of p21-3MR mouse model. (**a**) Schematic of the p21-3MR transgene. (**b**) P21 expression in vital organs from DOXO-treated mice. (**c**) Experimental design, control group were given 2 doses of PBS; the DOXO group were given 2 doses of DOXO (10 mg/kg); the DOXO+GCV group were given 2 doses of DOXO, GCV was given immediately after DOXO treatment, a total of 5 doses. (**d**) Initial body weights of p21-3MR mice from control/DOXO and DOXO+GCV group.

The p21-3MR mouse model harbors a transgene encoding a fusion protein promoted by senescence-sensitive p21. This transgene encodes three fusion proteins: luciferase (LUC), monomeric red fluorescent protein (mRFP), and herpes simplex virus thymidine kinase (HSV-TK) (Figure 1a). We administered DOXO to p21-3MR mice and collected and analyzed their vital organs 48 h later, including the heart, liver, spleen, lungs, kidneys, and adipose tissues to measure changes in p21 expression. This figure showed p21 expression in vital organs normalized to spleen expression (Figure 1b). Especially noteworthy are the results in adipose tissue, where the level of p21 was 33 times higher than that within the spleen. This suggests that adipose tissues are most sensitive to DOXO induction. We selected a subgroup of mice that received DOXO and simultaneously received GCV (Figure 1c). In visceral adipose tissue, 25 mg/kg was the best concentration for clearing p21^high^ cells and preventing p21 accumulation. Inguinal adipose tissue exposed to a 15 mg/kg dose exhibited the most favorable results, but p21 expression remained higher than with PBS. Our final decision was to set a minimum dose concentration of 15 mg/kg (Figure S1a). It is noteworthy that the body weight of the control group remained stable, whereas the DOXO mice lost significant body weight over time. However, the trend of body weight loss was alleviated after receiving GCV (Figure 1d). These results suggest that GCV may have a protective effect against the negative effects of DOXO-induced toxicity in the mice (Figure S1b).

### 2.2. GCV Treatment Selectively Kills p21^high^ Cells In Vivo

**Figure 2:**
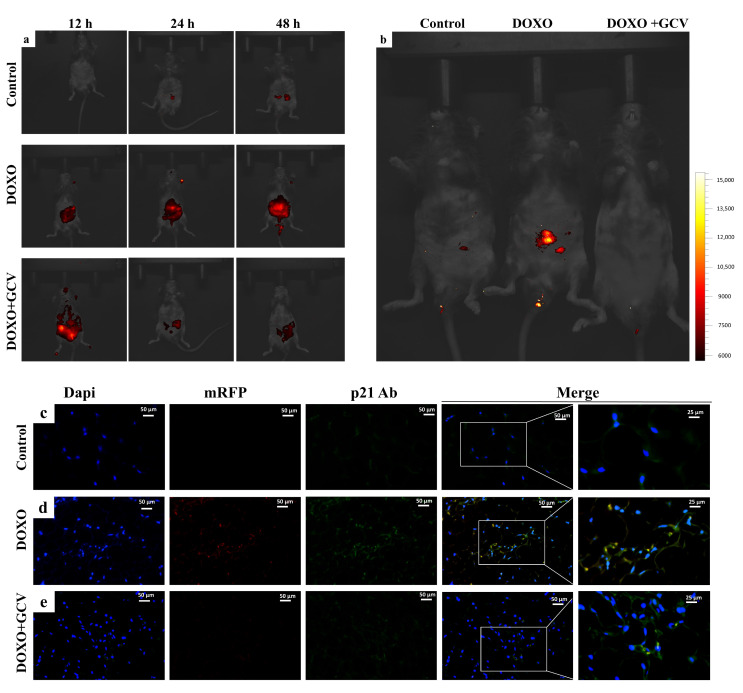
GCV treatment selectively kills p21^high^ cells in vivo. (**a**,**b**) Representative images of mRFP activity in the control/DOXO or DOXO+GCV treated groups. Mice were given 2 doses of DOXO (10 mg/kg) or PBS. GCV (15 mg/kg) was given immediately after DOXO treatment, a total of 5 doses. BLI was performed 12 h, 24 h, and 48 h after the administration. Experiments were repeated in 3 mice. (**c**) Representative micrographs of visceral fat sections from control/DOXO and DOXO+GCV group. Blue, dapi; red, mRFP; green, p21 ab(antibody). (**d**,**e**) P21 mRNA expression level of p21-3MR mice from control/DOXO and DOXO+GCV groups.

The p21-3MR mouse model allows us to detect p21^high^ cells in live mice through time-dependent bioluminescence imaging (BLI). Upon injection of PBS, no fluorescence was observed; however, the fluorescence signal increased significantly (particularly in the abdomen) within 48 h of DOXO injection. In the DOXO+GCV group, the strongest fluorescence was observed at 12 h following injection, and the luminescence trend was opposite to that of the DOXO group, decreasing by a significant amount over time (Figure 2a). We also observed faint fluorescence in the control group, likely due to blebs during injection of the luciferase substrate. The image demonstrates that the fluorescence intensity in the DOXO+GCV group was the same at 48 h as that in the PBS group, which means that GCV can be used to interfere with p21 gene expression in vivo and avoid p21^high^ cell accumulation (Figure 2b). By using BLI, we investigated how p21 transcriptional dynamics in vivo are observed. Because adipose tissue showed the greatest sensitivity to DOXO induction (Figure 1b), we chose it to validate the clearance effect of GCV. In the experiment, mRFP exhibited red fluorescence, while the p21 antibody displayed green fluorescence. The picture illustrates yellow fluorescence caused by the overlap of the two fluorescence in DOXO-induced p21 high-expression tissues, which proves that mRFP could report accurately with p21^high^ cells (Figure 2d). We observed minimal signals in either the control or DOXO+GCV group (Figure 2a,c), indicating that GCV reduces p21^high^ senescent cells in adipose tissue. This is further confirmed by mRNA for p21(Figure S2a). The same trend was observed in inguinal adipose tissue (Figure S2b). We observed a decrease in p16 expression after GCV administration, albeit less than that of p21. We speculate that this may be attributed to the clearance of p21 (Figure S2c).

### 2.3. GCV Counteracts the Expression of Senescence Markers in Vital Organs

**Figure 3:**
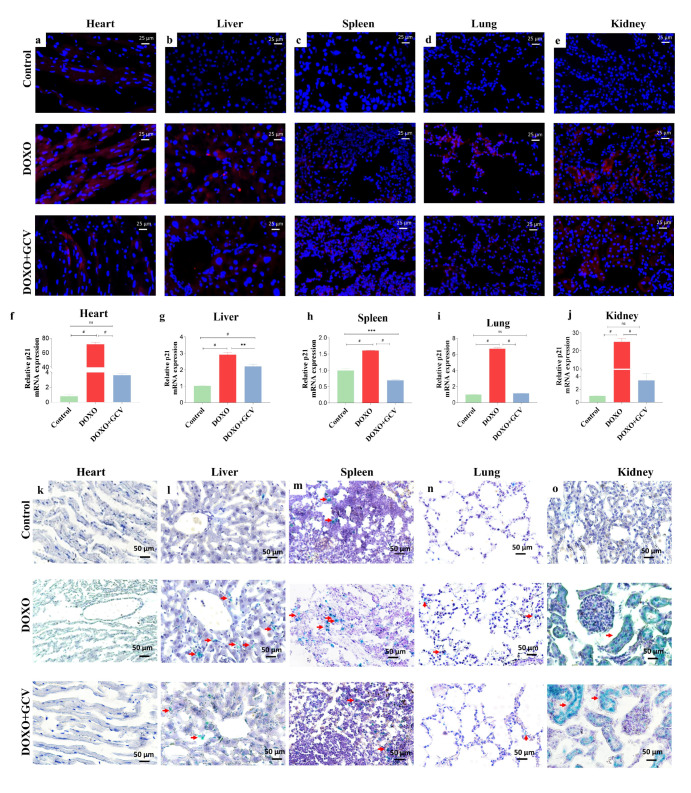
GCV counteracts the expression of senescence markers in vital organs. (**a**–**e**) Representative micrographs of 5 vital organs in p21-3MR mice from control/DOXO, and DOXO+GCV groups. (**f**–**j**) Representative p21 mRNA expression level of 5 vital organs in p21-3MR mice from control/DOXO, and DOXO+GCV groups. (**k**–**o**) Representative SA-β-gal activity of 5 vital organs in p21-3MR mice from control/DOXO, and DOXO+GCV groups. Red: mRFP, Blue: DAPI, Red arrow: senescent cells. **, *p* < 0.01. ***, *p* < 0.01. #, *p* < 0.0001, ns, no significant. Experiments were repeated in 3 mice. One-way ANOVA with Bonferroni correction for multiple comparisons.

We assessed the senescence process in vital organs including the heart, liver, spleen, lung, and kidney by examining the fluorescence intensity of mRFP (Figure 3a–e), transcript levels of p21 (Figure 3f–j), and SA-β-gal activity (Figure 3k–o). Treatment with DOXO induced significant senescence in all tissues except the spleen. Both heart and kidney tissues displayed intense fluorescence (Figure 3a,e). As compared to the control group, the DOXO group from both the heart and kidneys showed 71.5-fold and 24.9-fold higher levels of p21 mRNA expression (Figure 3f,j). The liver and lung also exhibited stronger fluorescence (Figure 3b,d) and GCV had a better therapeutic effect on senescence in the lung, resulting in p21 expression levels that were not significantly different from the control group (Figure 3g,i). The liver and spleen showed relatively smaller differences in p21 expression than other tissues (Figure 3g,h). Additionally, we performed SA-β-gal staining, a common marker for senescence. We observed that both heart and kidney exhibited a large green range in this part of the experiment, which is consistent with the trend of mRFP and mRNA (Figure 3k,o). The only group that showed lower SA-β-gal-positive cells than controls in the DOXO+GCV treatment was the spleen (Figure 3m). GCV demonstrated excellent clearance throughout all tissues, particularly in the lungs. These suggest that the use of GCV to interfere the process of DOXO-induced senescence is quite effective.

### 2.4. Clearance of p21^high^ Cells Improves Histological Changes in Vital Organs

**Figure 4:**
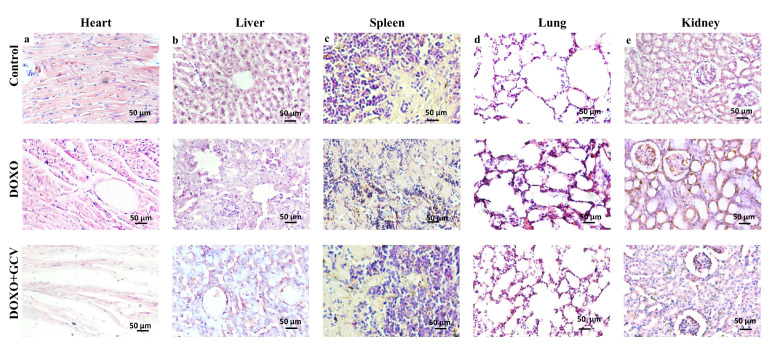
Histological changes in vital organs following GCV administration. (**a**–**e**) Representative HE staining of 5 vital organs in p21-3MR mice from control/DOXO, and DOXO+GCV groups.

DOXO led to typical inflammatory/necrotic lesions in histological tissues in mice. Heart: histopathological changes in the DOXO-treated group included degeneration and death of cardiomyocytes, accompanied by infiltration of inflammatory cells, and myofibrillar loss. H&E staining of sections in the DOXO-treated group revealed myocardial injury with cardiomyocyte vacuolization, a hallmark of DOXO-induced cardiotoxicity. It is worth emphasizing that GCV exacerbated the adverse cardiac histological changes; mice receiving GCV injections suffered more myofibril loss (Figure 4a).

Liver: The control group showed typical liver morphology and histological architecture. Inflammatory cell infiltrate around the sinusoids, focal necrosis, and degeneration of hepatocytes are some of the common histopathological changes observed in the liver tissue of the DOXO group. Compared to the DOXO group, sections from the DOXO+GCV group showed slightly less degeneration of hepatocytes and focal necrosis (Figure 4b). 

Spleen: the DOXO group saw an increase in white pulp, which was observable as expanded white pulp areas under microscopy in comparison to the control group, which had a precise splenic organization with distinct regions of red and white pulp. We did not observe significant inflammatory infiltration in the spleen in any of the groups (Figure 4c).

Lung: Mice treated with DOXO alone exhibited severe thickening of the alveolar wall by proliferation pneumocytes with concurrent reduced alveolar spaces. This is likely due to damage to the epithelial cells that line the alveoli, leading to inflammation and scarring of the lung tissue. The changes were abrogated by GCV (Figure 4d).

Kidney: Administration of DOXO revealed focal inflammatory cell infiltration in the cortical portion, with congestion of blood vessels, focal hemorrhage, and degeneration in the tubular lining epithelium. We found that GCV restored the morphology of the kidney (Figure 4e).

### 2.5. SASP Factor Changes in Vital Organs following GCV Administration

**Figure 5:**
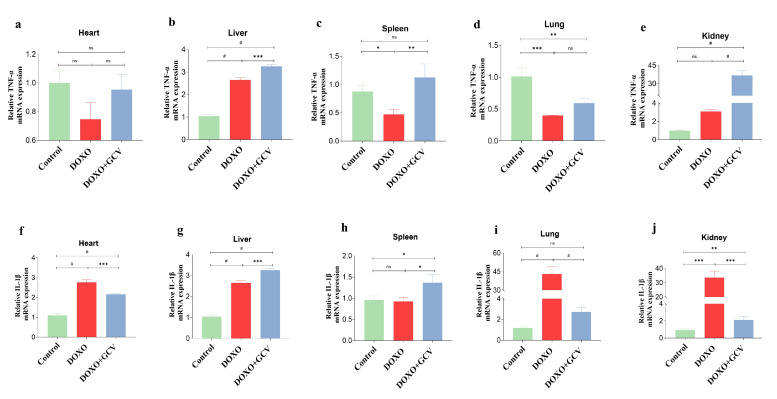
SASP factor changes in vital organs following GCV administration. (**a**–**e**) Representative TNF-α mRNA expression level of 5 vital organs in p21-3MR mice from control/DOXO, and DOXO+GCV groups. (**f**–**j**) Representative IL-1β mRNA expression level of 5 vital organs in p21-3MR mice from control/DOXO, and DOXO+GCV groups. IL-1β: interleukin-1 beta; TNF-α: tumor necrosis factor-alpha. *, *p* < 0.1; **, *p* < 0.01; ***, *p* < 0.01; #, *p* < 0.0001; ns, no significance. One-way ANOVA with Bonferroni correction for multiple comparisons.

We examined the impact of GCV against DOXO-mediated increase in the biomarkers of senescence, including TNF-α (Figure 5a–e) and IL-1β (Figure 5f–j). The results revealed that mice treated with DOXO alone exhibited a marked increase in the levels of pro-inflammatory biomarker IL-1β, which was most prominent in the lungs and kidney (Figure 5i,j). Despite its ability to clear senescent cells, GCV does not appear to reduce inflammation levels, but rather exacerbates the level of TNF-α in the liver and spleen (Figure 5b,c). Specifically, we found that the spleen was the tissue with the least significant level of inflammation after DOXO administration, consistent with the previous results. Due to the notable variations in TNF-α expression across different organs, we conducted gradient concentrations of GCV on TNF-α in visceral and inguinal adipose tissues. Our results demonstrated a stronger association between TNF-α and p21 in adipose tissue (Figure S3). Nevertheless, additional experimental investigations are required to further examine these findings.

## 3. Discussion

P21 represents one of the most studied transcriptional targets of p53, the mammalian cyclin-dependent kinase inhibitor family [25,26]. Our study developed and validated a new mouse model to explore the disease process by understanding the spatial and temporal distribution of p21^high^ cells. This mouse strain is capable of (a) detecting senescent cells in living animals, (b) labeling senescent cells in mouse tissues, and (c) eradicating senescent cells with a compound that is otherwise ineffective in wild-type mice. Previous studies involving the p21-3MR mouse have not been reported, so multiple validation studies must be performed to guarantee that rigorous studies are conducted.

The p21-3MR mice were treated with DOXO and determined whether we were able to identify p21^high^ cells in vivo. We used mRNA expression to verify the expression level of p21 in each tissue. Taking into account that even the same type of stress triggers different levels of senescence in different cell types, this is another manifestation of heterogeneity, which enables us to adjust the protocol in clinical practice accordingly to avoid harming vital organs outside of the target organ. Our result agrees with the study carried out by Xu et al., in the sense that adipose tissue was the most sensitive following the application of DOXO treatment [8]. We also found that p21^high^ cells have a causal role in drug-induced physical weakness, which manifested in a sharp decline in body weight and disruption of systemic homeostasis. GCV prevented this to a certain extent, but not completely.

We examined the entire body of p21-3MR mice for mRFP fluorescence in order to distinguish tissues experiencing cellular senescence, whole-body imaging results showed excessive abdominal fluorescence in the DOXO group, which is a result of high p21 expression in visceral adipose tissue. The second step involved examining adipose tissue sections for the presence of both mRFP and antibodies to p21 to determine whether mRFP could serve as a surrogate marker for p21. Compared to wild-type mice, p21-3MR mice are superior in that they can be used to observe the presence of p21^high^ cells directly through fluorescence microscopy with less interference by an autofluorescent background, which makes them highly suitable for in vivo fluorescence imaging. Both results confirm that this model can be effectively used to track p21^high^ cells. It should be noted that we were unable to identify specific values of p21 expression in tissues using tissue fluorescence and had to combine it with other methods to achieve this goal.

The use of DOXO in the treatment of various types of cancer has made it one of the most widely used chemotherapeutic agents, but it induces dose-dependent multisystem toxicity, particularly cardiovascular damage [27]. Transcriptome analysis indicated that cardiotoxicity induced by acute DOXO treatment was associated with dysregulation of the expression of genes associated with the p53 pathway [24,28]. It has been suggested that this may be achieved through cell-cycle arrest. We have previously shown that intraperitoneal administration of GCV eradicated p21^high^ cells in all tissues, not only adipose tissues. Aside from the expression of p21, we used SA-β-gal activity to estimate the senescence rate in major organs. In conjunction with the histological analysis, it appears that removing p21^high^ cells in the short term had a positive therapeutic effect in almost in every tissue. The morphological destruction of the tissue was not completely reversed, which may also be related to the period of treatment, considering that the level of p21 expression in most tissues remained high by the end of the experiment.

The p21 gene is not just involved in maintaining cell-cycle arrest, it plays an important role in establishing SASP through *retinoblastoma* protein (Rb)-dependent transcription [29]. According to a recent report, DOXO induced inflammation through the upregulation of the production of inflammatory cytokines (TNF-α and IL-β) [30,31]. We observed that DOXO treatment enhanced the secretion of IL-1β, especially in the lung and kidney. This is consistent with the former study [32]. We also found that the levels of TNF-α were not significantly altered in the heart, spleen, and lung after DOXO administration. The reason for this may be that the dose or concentration was not sufficient to induce high expression in these tissues. The level of TNF-α was increased in the liver, the spleen, and especially in the kidney following the administration of GCV treatment, which may be the result of liver-related toxicity of HSV-TK/GCV [33].

During tumor treatment, chemotherapeutic agents/radiotherapy can induce cellular senescence [34,35]. Previous studies suggest that therapy-induced senescence (TIS) correlates with positive treatment results and could be an index of therapeutic efficacy [36]. Currently, some progress has been made regarding the treatment of TIS. For example, targeted removal of senescent cells can prevent cancer recurrence and complications, enhance healthy lifespan, and possibly extend the life expectancy of cancer patients [35]. Our study utilized the senescent cell clearance function of transgenic mice and found that the clearance of the p21 senescent cell population in addition to p16 senescent cells resulted in better treatment of DOXO-induced toxicity, especially in the lung. This has not been reported in the p16-3MR mouse before. The main alteration in the lung observed in our results after DOXO administration was fibrosis. Elevated p21, as a characteristic of fibroblast senescence resulting in fibrotic lesions, has been extensively studied [37,38], especially in chronic obstructive pulmonary disease (COPD) and idiopathic pulmonary fibrosis (IPF) [39,40]. We hypothesize that the better outcome achieved in the lungs in our results is also related to this point.

Single-cell tracking and experimental cellular research has unraveled heterogeneity in drug-induced senescence [41]. These studies point to the appearance of p21 in the early stages of TIS and determine proliferation–senescence cell fate after chemotherapy. In tumors, the P21^high^ cell is a double-edged sword, in that transient inhibition of p21 may be therapeutically beneficial. However, insufficient induction of p21 expression may actually lead to tumor growth [42,43]. Therefore, further study of the kinetics of p21 production in tumors could offer a novel and promising treatment strategy [44]. Our mouse model presents a useful approach for testing the toxicity of anti-cancer drugs and investigating the role of senescent cells in tumors. Our findings suggest that the model may have the potential to contribute to the advancement of cancer research and drug development through assessing drug efficacy and optimizing dosing regimens based on tumor size and response. Moreover, targeting specific types of senescent cells in tumors may lead to the development of more effective therapies. By providing researchers with a comprehensive understanding of tumor biology and drug effects, our model can contribute to ongoing efforts to improve cancer treatment outcomes.

The limitation of this study was the fact that only two SASP factors, TNF-α and IL-1β, were measured. Thus, our future studies will examine transcriptomics of senescence and other markers for a more comprehensive analysis. This mouse is a powerful tool for studying the interaction between p21 and SASP factors and various signaling pathways. Further, the use of two genetic mice, p21-3MR and p16-3MR, provides a way to directly compare the effects exerted by the two senescent pathways and may enable more accurate results. It should be noted that GCV only serves as a means of clearing p21^high^ cells and does not reduce the inflammatory response. Even though this study has demonstrated the beneficial effects of p21^high^ cell clearance in many tissues, it focuses on only one perspective, and the deeper mechanisms remain unclear and require further investigation.

## 4. Materials and Methods

### 4.1. Animals

To generate parental heterozygous mice, we used CRISPR-Cas9 technology to inject the exogenous gene 3MR into C57/BL mouse embryos. In collaboration with Shanghai Nanmo Biological Co. (Shanghai, China), we successfully designed and constructed p21-3MR transgenic mice. Animals were maintained in specific pathogen-free (SPF) cages and provided standard food and water. Humane care was provided to each animal during the experiments according to the criteria outlined in the Guide for the Care and Use of Laboratory Animals published by the National Institutes of Health (NIH).

### 4.2. Drug Treatments

For DOXO treatment, mice were given DOXO (Sigma, St. Louis, MO, USA) at a dose of 10 mg/kg, once daily for 2 consecutive days via one intraperitoneal injection per day, and 1 dose of GCV(15 mg/kg) was given at the same time as DOXO administration for 5 days during the experiment.

### 4.3. Fluorescence Microscopy in Tissue Section

Fluorescence imaging was employed to determine mRFP expression in the heart, liver, spleen, lung, kidney, inguinal fat, and visceral fat from the control, DOXO, and DOXO+GCV group. More specifically, these organs were inflated with a 50% solution of optimal cutting temperature (OCT) compound and 10 μm frozen sections were cut using a rotary microtome-cryostat. Immediately after sectioning, samples were fixed and mounted using Prolong (Cat# P36962, Life technologies Corporation, Eugene, OR, USA) with DAPI. Images were acquired using a Nikon Eclipse Ni-U fluorescence microscope at 200× magnification using Advance SPOT software.

### 4.4. Immunofluorescence Staining

All sections were blocked with 10% goat serum (Maxim) and then incubated with a primary antibody at 4 °C overnight and with a secondary antibody for 1 h at 37 °C. Slides were then stained with DAPI (Sigma, St. Louis, MO, USA) and mounted with an anti-fade reagent (Invitrogen, Waltham, MA, USA). The antibodies used in this experiment were as follows: pp21 (1:100, Abcam, Cambridge, UK) and Alexa Fluor 488 donkey anti-rabbit IgG (H+L) (1:1000, Invitrogen, Waltham, MA, USA). Images were acquired with a Nikon DS-Ri1 microscope.

### 4.5. RNA Extraction and Quantitative Real-Time PCR

Total RNA was extracted using the RNAiso Plus reagent (Takara Bio, Shiga, Japan), reverse transcribed using a PrimeScript™ RT reagent kit with gDNA Eraser (Perfect Real Time) (Takara Bio, Shiga, Japan), and then subjected to qPCR analysis using SYBR Mix and a LightCycler System (Roche, Basel, Switzerland). Fold changes of mRNA were calculated by the 2−ΔΔCt method after normalization to the expression of GAPDH.

### 4.6. Bioluminescence Imaging

Mice were anesthetized with isoflurane gas (Piramal Critical Care, Bethlehem, PA, USA) and injected intraperitoneally with 3 mg D-luciferin (Gold Biotechnology, St. Louis, MO, USA) in 200 μL PBS. Five min after injection, mice were placed in IVIS spectrum in vivo imaging system (PerkinElmer, Waltham, MA, USA) and bioluminescence images were subsequently captured with a 3 min exposure time. Regions of interest (ROIs) were manually selected in a consistent way for individual mice in same cohort. The bioluminescent signal of the ROI was calculated using Living Image 4.5.5 software (PerkinElmer).

### 4.7. Senescence-Associated β-Galactosidase Activity

SA-β-gal staining was performed using an SA-β-gal staining kit (Beyotime, Shanghai, China) according to the manufacturer’s instructions. To identify the proportion of SA-β-gal-positive cells, hematoxylin (Beyotime, Shanghai, China) staining was also performed, and digital images of ten randomly selected fields were acquired using a microscope (Nikon DS-Ri1, Tokyo, Japan).

### 4.8. Histological Analysis

All tissues were fixed in 4% PFA (Sigma-Aldrich, St. Louis, MO, USA) for 48 h at 4 °C and were analyzed by H&E staining (Biotech Well, Shanghai, China)

### 4.9. Statistical Analysis

The data are expressed as the mean ± s.e.m. The significance of differences between the experimental groups was determined by one-way ANOVA with Bonferroni correction for multiple comparisons using SPSS 24.0. Values of *p* < 0.05 (two-tailed) were considered statistically significant (*). The bar chart was produced using GraphPad Prism 8 software.

## 5. Conclusions

In summary, our findings demonstrate the possibility of using p21-3MR reporter mice to estimate endogenous p21 expression in normal conditions and under pharmacologic toxic stress. We found that removing p21^high^ cells had a therapeutic effect on TIS in vital organs, as shown by a decrease in the levels of p21 and SA-β-gal, as well as changes in the SASP profile. As far as we are aware, this is the first attempt to utilize p21-3MR as a senescence model in pharmaceutical pathologies. Research involving the p21-3MR model will be necessary in future to determine how p21^high^ cells contribute to senescence-induced diseases.

## Data Availability

All data included in this study are available upon request by contact with the corresponding author.

## Supplementary Materials

The following supporting information can be downloaded at: https://www.mdpi.com/article/10.3390/ijms24065565/s1.
