# Supplementary material for: Generation of a p21 Reporter Mouse and Its Use to Identify and Eliminate p21high Cells In Vivo
Source: Int J Mol Sci. 2023 Mar 14;24(6):5565. doi: 10.3390/ijms24065565 (PMC10051249; doi:10.3390/ijms24065565)
Supplement: Supplementary file 1 [file ijms-24-05565-s001.zip › ijms-2204474-supplementary.pdf]

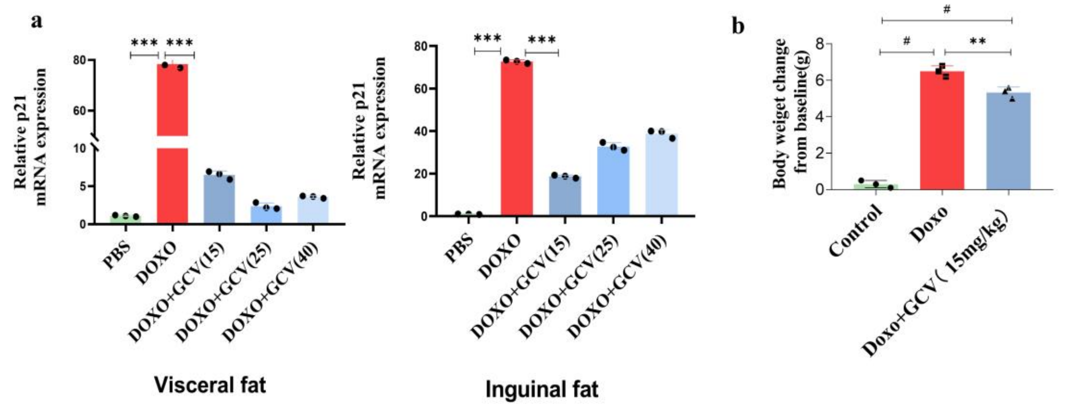

Figure S1: (**a**) P21 mRNA expression level of p21-3MR mice from Control/Doxo, and Doxo + GCV(15mg/kg), Doxo + GCV(25mg/kg), Doxo + GCV(40mg/kg) groups. (b) body weights change of p21-3MR mice from Control/Doxo, and Doxo + GCV group. **， P<0.01 ; ***， P<0.001; #, P<0.0001. One-way ANOVA with Bonferroni correction for multiple comparisons.

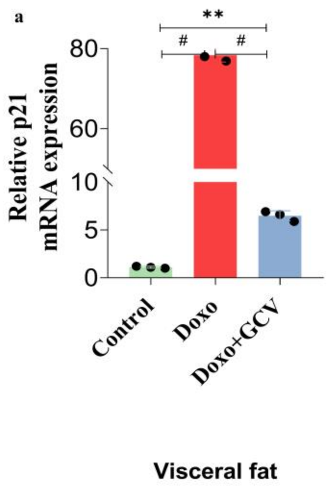
a
Relative p21 mRNA expression
**
#
#
80
60
10
5
0
Control
Doxo
Doxo+GCV
Visceral fat

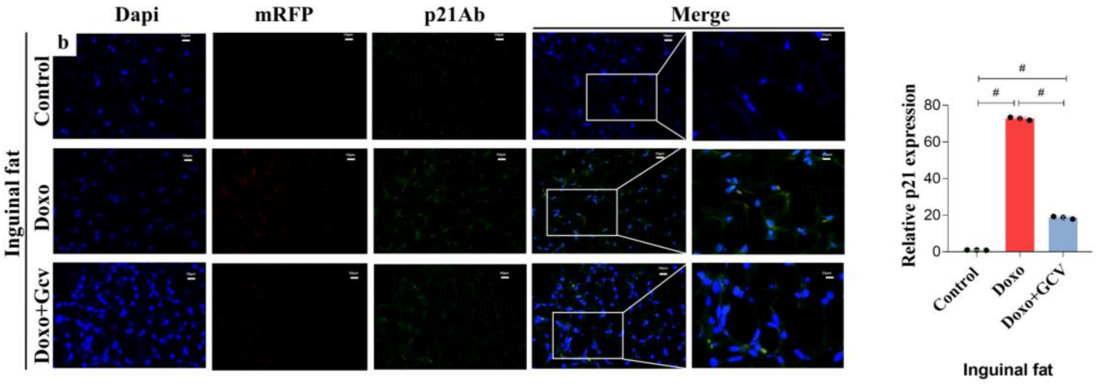
Dapi
mRFP
p21Ab
Merge
b
Inguinal fat
Control
Doxo
Doxo+Gcv
Relative p21 expression
#
#
#
80
60
40
20
0
Control
Doxo
Doxo+GCV
Inguinal fat

c

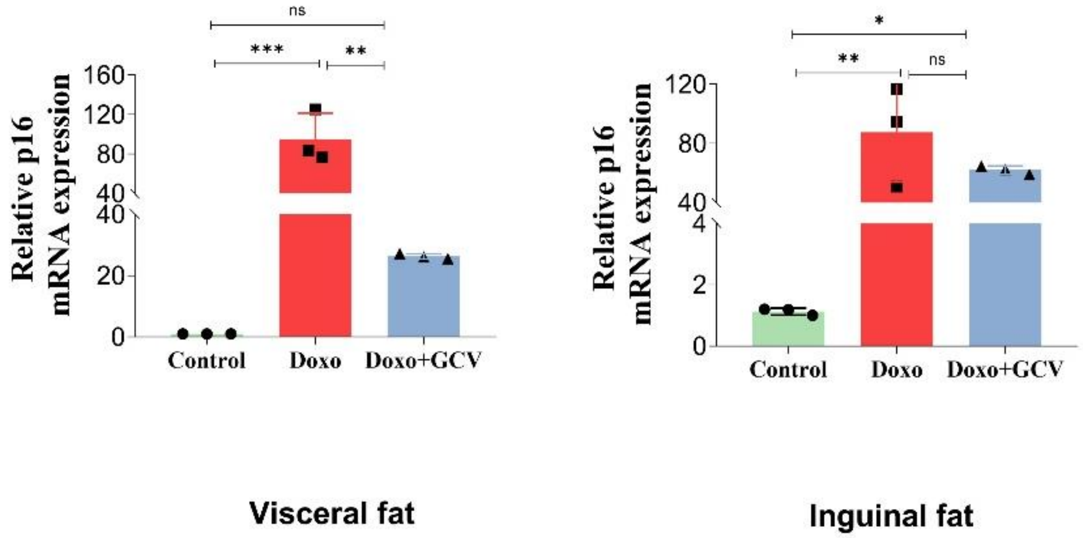
ns
***
**
Relative p16 mRNA expression
160
120
80
40
40
20
0
Control
Doxo
Doxo+GCV
Visceral fat
*
**
ns
Relative p16 mRNA expression
120
80
40
4
2
0
Control
Doxo
Doxo+GCV
Inguinal fat

Figure S2. a: P21 mRNA expression level of p21-3MR mice from Control/Doxo, and Doxo + GCV groups. **， P<0.01 #, P<0.0001. One-way ANOVA with Bonferroni correction for multiple comparisons. **b**: (left)Representative micrographs of inguinal fat sections from Control/Doxo, and Doxo + GCV group. Blue, dapi; red, mRFP; green, p21ab(antibody); (right) p21 mRNA expression level of p21-3MR mice from Control/Doxo, and Doxo + GCV groups. Representative images of n = 4–5/group were provided. Blue, Dapi; red, mRFP; green, p21ab(antibody). **， P<0.01 #, P<0.0001. One-way ANOVA with Bonferroni correction for multiple comparisons. **c** P16mRNA expression level of p21-3MR mice from Control/Doxo, and Doxo + GCV groups. *， P<0.1;**， P<0.01; ***， P<0.001 #, P<0.0001;ns, no significant. One-way ANOVA with Bonferroni correction for multiple comparisons.

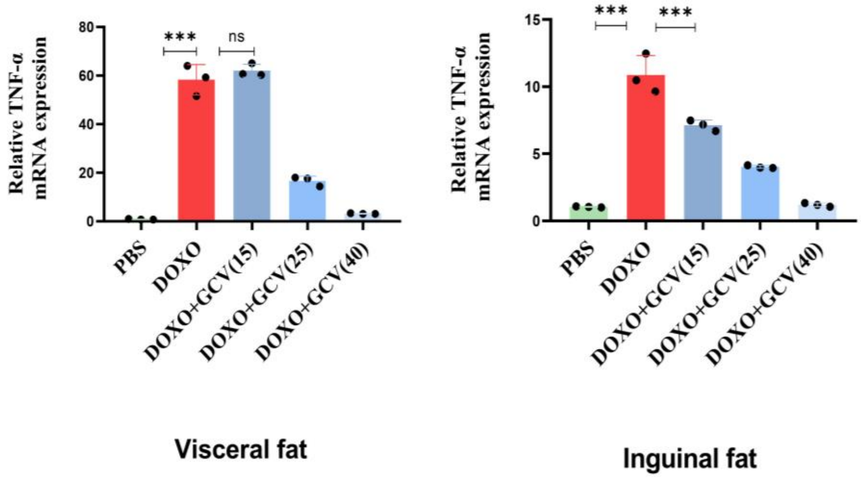

Figure S3. TNF-α mRNA expression level of p21-3MR mice from Control/Doxo, and Doxo + GCV(15mg/kg), Doxo + GCV(25mg/kg), Doxo + GCV(40mg/kg) groups. ***， P<0.001; ns, no significant. One-way ANOVA with Bonferroni correction for multiple comparisons.
